# Safety Challenges of Using High Dose Baclofen for Alcohol Use Disorder: A Focused Review

**DOI:** 10.3389/fpsyt.2018.00367

**Published:** 2018-08-22

**Authors:** Benjamin Rolland, Nicolas Simon, Nicolas Franchitto

**Affiliations:** ^1^Service Universitaire d'Addictologie de Lyon (SUAL), Pôle MOPHA, CH Le Vinatier, Bron, France; ^2^Univ Lyon, Inserm U1028, CNRS UMR5292, UCBL, CRNL, Lyon, France; ^3^APHM, INSERM, IRD, SESSTIM, Hop Sainte Marguerite, Service de Pharmacologie Clinique, CAP-TV, Aix Marseille Univ, Marseille, France; ^4^Inserm, UMR1027, Université de Toulouse, UPS, Toulouse, France

**Keywords:** baclofen, alcohol use disorder, safety, dosing preferences, tolerability, public health

## Abstract

Since the early 2000s, the gamma-aminobutyric acid type B (GABA-B) receptor agonist baclofen has been extensively used for treating alcohol use disorder (AUD). In some countries, like France, Australia, or Germany, baclofen has been used at patient-tailored dose regimens, which can reach 300 mgpd or even more in some patients. The GABA-B-related pharmacology of baclofen expose patients to a specific profile of neuropsychiatric adverse drug reactions (ADRs), primarily some frequent sedative symptoms whose risk of occurrence and severity are both related to the absolute baclofen dosing and the kinetics of dose variations. Other frequent neuropsychiatric ADRs can occur, i.e., tinnitus, insomnia, or dizziness. More rarely, other serious ADRs have been reported, like seizures, manic symptoms, or sleep apnea. However, real-life AUD patients are also exposed to other sedative drugs, like alcohol of course, but also benzodiazepines, other drugs of abuse, or other sedative medications. Consequently, the occurrence of neuropsychiatric safety issues in these patients is essentially the result of a complex multifactorial exposure, in which baclofen causality is rarely obvious by itself. As a result, the decision of initiating baclofen, as well as the daily dose management should be patient-tailored, according the medical history but also the immediate clinical situation of the patient. The overall safety profile of baclofen, as well as the clinical context in which baclofen is used, have many similarities with the use of opiate substitution medications for opiate use disorder. This empirical statement has many implications on how baclofen should be managed and dosing should be adjusted. Moreover, this constant patient-tailored adjustment can be difficult to adapt in the design of clinical trials, which may explain inconsistent findings in baclofen-related literature on AUD.

## Introduction

Baclofen is an agonist of the gamma aminobutyric acid type B (GABA-B) receptors. In the early 1970s, baclofen has been labeled for neurological states spasticity, which may occur in severe neurological injuries or some neurological diseases like multiple sclerosis (1). In these neurological indications, baclofen is used orally, or is directly infused within the central nervous system via intrathecal pumps. In its oral labeled use, the maximum approved dosing is generally 80 mg per day (mgpd) for outpatients, and 120 mgpd for inpatients.

From the beginning of the 2000s, an extensive number of clinical studies have assessed the efficacy of baclofen in the treatment of alcohol use disorder (AUD). Until recently, these studies had essentially focused on low-dose baclofen, i.e., a maximum dosing of 30 mgpd or more rarely 60 mgpd. So far, the results of these efficacy studies have been relatively contrasting (2), even if recent meta-analyses have found a significant effect of baclofen in AUD, either for maintaining abstinence (3) or reducing drinking (4). In parallel, an empirical use of baclofen for AUD has also progressively spread among clinicians in several countries, for example France (5–7) or Australia (8). Due to its off-label nature, however, this use has bloomed into very heterogeneous practices, particularly with regard to the prescribing schemes and patterns of dose used (9).

Baclofen prescribing practices usually vary according to the underlying rationale of prescribers. Using baclofen in AUD has occasionally been supported by a possible action of baclofen on the dopaminergic transmission in the nucleus accumbens, which could thereby reduce craving and alcohol use in subjects with AUD (10). Another approach is that ethanol effects on the brain could in part be mediated by an action on the GABAB receptors. As baclofen can prevent the occurrence of alcohol-related withdrawal symptoms, some other authors have hypothesized that baclofen could be a form of substitution treatment for alcohol (11, 12), thus justifying that baclofen could be used in AUD similarly to how methadone or buprenorphine are used of opiate use disorder (13).

This latest rationale implies a dose-effect relationship, but also the fact that baclofen dosing should be adjusted on a patient-tailored manner (9, 14). This has been one of the arguments for using high doses in some patients. Unfortunately, the only three clinical trials that have explored the efficacy of very high doses in subjects with AUD have also yielded contradictory results with two negative trials (15, 16), and two other trials finding a significant difference compared to placebo (17, 18). This may explain why meta-analyses have not found any dose-effect relationship so far (3, 4).

In France, the empirical use of baclofen has largely spread from 2007 to 2013, when the estimated number of treated patients had reached 200,000 subjects (19). Though baclofen use has substantially decreased since 2014 in France, an important collection of safety data have been gathered, analyzed, and published, with regard to the safety profile of baclofen in the specific population of AUD patients. This French experience, mixed with other international studies, thus represent a rich amount of safety data about baclofen use in real-life patients with AUD, including at high doses, even if the prescribing habits and protocols can vary according to places and teams (9). This focused narrative review thus addresses the main pharmacoepidemiological and pharmacovigilance studies published on the off-label use of baclofen for AUD.

## Adverse drug reactions with demonstrated causality of baclofen

### Sedation and related consequences

Sedation, and the related consequences, like dizziness or confusion, are by far the most frequent ADRs related to baclofen (see Table 1). Moreover, the level of sedation has been found correlated with baclofen dosing and dose increases. In the three randomized clinical trials (RCTs) using high dose baclofen, sedation was reported by between 38.0 and 46.6% of the patients treated with high dose baclofen (15, 16, 18) vs. 22.6% among patients treated with low-dose baclofen (15), and between 17.7 and 25% in patients treated with placebo. Whereas no serious ADR was reported in relation to baclofen-induced sedation in the first two RCTs, only two cases of fall were reported in Reynaud et al. (16) (See Table 1). In the French national pharmacovigilance database, sedation/drowsiness was reported in 24.5% of all types of reported ADRs (see Table 1), and 32% of all cases of non-serious ADRs (20). Similarly, 17.4% of all notifications with serious ADRs reported confusion, while 11.5% of them reported sedation, and 9.6% of them reported coma (Table 2). Baclofen main safety concerns are thus by far the consequences of baclofen-induced sedation.

**Table 1:** Main ADRs occurring in AUD patients treated with high dose baclofen.

	**Occurrence rates in RCTs (HDB vs. placebo)^a^**	**Proportion of all ADRs reported in the FPVD^b^**
Sedation/Dizziness/Fatigue	38.0 to 46.6% vs. 17.7 to 25%	24.5%
Insomnia/Sleep Disorders	32.1 to 38.8% vs. 14.3 to 30.8%	7%
Headache	26.7% vs. 15.0%	–
Paraesthesia	14.4 to 16.6% vs. 3.6 to 4.4%	–
Restlessness/Fasciculations	10.8 to 14.3% vs. 3.6 to 3.8%	3.8%
Headache	24 (15.0%) 42 (26.7%)	
Dry mouth	7.6 to 20.7% vs. 1.6 to 5.0%	10%

a *(15, 16, 18)*.

b *(20)*.

*ADRs, adverse drug reactions; AUD, alcohol use disorder; RCTs, randomized controlled trials; HDB, high-dose baclofen; FPVD, French Pharmacovigilance Database*.

**Table 2:** Ten most frequent serious ADRs reported in the FPVD in patients treated with baclofen for AUD.

	**Proportion of all serious ADRs reported in the FPVD (%)**
Confusion	17.3
Seizures	11.5
Major sedation	11.5
Agitation	10.9
Coma	9.6
Hallucinations	7.7
Falls	7.1
Behavioral disorders	5.8
Baclofen withdrawal syndrome	5.1
Space-time disorientation	5.1

Auffret et al. (20).

*ADRs, adverse drug reactions; AUD, alcohol use disorder; FPVD, French Pharmacovigilance Database*.

In all these data, however, the role of alcohol or other drugs was rarely studied. Even if baclofen can definitely induce sedation, patients with AUD are likely to experience other causes of sedation. In particular, it has been found that the main predictor of major sedation in AUD patients treated with baclofen is the concurrent level of alcohol use (21). Consequently, the occurrence of severe sedation or coma among AUD subjects treated with baclofen is probably the result of a complex equation which integrates baclofen dosing and recent dose increases, but also the level of alcohol use, and other drugs of abuse or sedative medications that may be more or less regularly ingested by the subject. In practice, the exact causality of baclofen in a situation of severe sedation should thus be addressed on a case-by-case basis, using an exhaustive anamnesis, and a rigorous pharmacovigilance approach (22). The mechanisms through which baclofen can induce or participate in inducing sedation, are probably in link with the specific pharmacology of the GABA-B receptor, which is known for regulating the activity of the GABA-A receptors (23, 24).

Similarly, the diverse toxicology studies that have addressed baclofen involvement in the severity of intoxications, including self-poisoning and resuscitation hospitalizations, have found that many pharmacological cofounders may actually contribute to the overall severity and the clinical outcomes (25–27). Consequently, baclofen should only be considered as one of the many contributing severity factors of self-poisoning and drug intoxication in AUD patients (28). Moreover, these situations of intoxications more specifically affect some subpopulations of AUD subjects, in particular those who are more likely to display self-poisoning and suicide attempts. This is particularly the case for subjects with personality disorders, including borderline personality disorder, in whom the frequency and severity of poly-substance and poly-drug intoxications have been found particularly high (29). This can also apply to AUD subjects with comorbid borderline personality disorder who are treated with baclofen (30). However, it is unclear whether these increased situations of self-harm and their consequences can be accounted directly on baclofen.

### Other neuropsychiatric ADRs induced by baclofen

Other neuropsychiatric symptoms can occur in patients with baclofen, with a demonstrated causality of the drug. This is in particular the case for insomnia (20), tinnitus (20, 31), and more rarely, seizures (32, 33), hallucinations (20, 34), and manic symptoms (35). In addition, baclofen withdrawal is associated with a specific withdrawal syndrome that is addressed in section Baclofen withdrawal syndrome and non-neuropsychiatric baclofen-induced side effects. In the RCTs using high-dose baclofen, insomnia or sleep disorders affected between 32.1 and 38.8% of the patients treated with high-dose baclofen, vs. between 14.3 and 30.8% of those receiving placebo (16, 18).

The mechanisms through which baclofen can trigger these different types of neuropsychiatric symptoms are relatively unclear. Animal studies have found that baclofen could enhance the serotonin and noradrelin levels in some parts of the brain (36–38). This could contribute to baclofen anxiolytic properties (39), but also to a possible antidepressant effect that still remains to be more clearly demonstrated (40). This could also explain the risk of developing manic symptoms (40). The complex effects that baclofen may have on sleep could result from similar mechanisms. In addition, the regulation of the pineal gland is influenced by the GABAergic signaling, and it involves the GABA-B receptors (41).

Concerning the risks of both seizures and tinnitus, it is interesting to note that, depending on the situation, the level of activity of the GABA-B receptors could have both pro- or anticonvulsant consequences, (42), and pro- and anti-tinnitus effects (43). This seems to highlight that the GABA-B receptors have a complex modulatory role of other receptors involved in these symptoms, in particular the GABA-A receptors. Indeed, both the activity and the composition of the GABA-A receptors are directly regulated by GABA-B receptors (23, 24, 44, 45). Similarly, it could also explain why baclofen can cause insomnia in some patients, and sedation in other patients. Finally, baclofen was recently found to induce or increase the severity of central sleep apnea in AUD patients (46). This clinical finding is in line with previous animal studies that found that baclofen decreased the firing rate of respiratory neurons in the mesencephalon (47).

### Baclofen withdrawal syndrome and non-neuropsychiatric baclofen-induced side effects

Baclofen withdrawal can induce a specific withdrawal syndrome, which consists of irritability, confusion, seizures, hallucinations, and noradrenergic peripheral symptoms. In subjects with AUD, baclofen withdrawal syndrome can easily mistaken for alcohol withdrawal syndrome. (48). Moreover, benzodiazepines could be less effective for treating baclofen withdrawal syndrome than for treating alcohol withdrawal (48), and the reintroduction of baclofen seems to be the most appropriate therapy when possible. As of now, it is unknown at what daily dosing the risk of withdrawal syndrome may occur in the treated patients, and what are the other vulnerability features for experiencing baclofen withdrawal syndrome.

Diverse non-serious gastro-enteric ADRS, such as diarrhea, gastric pain, are frequently reported by AUD patients treated with baclofen (49, 50), even if baclofen causality was never properly explored for these specific types of symptoms. By contrast, baclofen causality was demonstrated in the occurrence of ankle edema, which seem to occur in approximately 5% of the AUD patients treated with baclofen (51). The mechanism through which edema can occur is relatively unclear, and might be related to a vasogenic effect of baclofen.

## Adverse drug reactions with unclear baclofen causality

The sedative effects of baclofen may theoretically expose the treated patients to an increased risk of intoxication and coma. This has thus raised some logical concerns about an increased risk of death in case of baclofen intoxication. It has been previously found that beyond 150 or 200 mg of baclofen intake, the severity of intoxication may require that the treatment be carried out in intensive care units (32, 52). Moreover, anecdotal reports have suggested that baclofen could be a factor of immediate increased risk for death in case of self-poisoning (53). However, in this case report, baclofen causality was not explored and many other potential factors could actually explain the death of the patient (22). Several French toxicological reports have also raised concerns about the vital risks of using baclofen in AUD patients, especially in case of intoxication (26, 27).

In July 2017, an internal study was conducted by the French Drug Agency and the French Health Insurance using the data of the French claims database. The authors of this study found that the chronic average baclofen dose range was significantly associated with the overall risk of mortality among AUD patients (54). These findings have thus questioned the safety of a chronic prescription of high doses of baclofen among AUD patients. However, this study, which has never been peer-reviewed and never published so far, has raised a lot of criticisms in France for different reasons, including the fact that the analyses were not controlled for most of the usual cofounders, starting with the concurrent level of alcohol use, which might be an obvious cause of mortality in these patients, and is frequently associated with the concomitant use of high doses of baclofen. At this stage, there is thus no definite evidence that using baclofen in AUD patients expose them to a dose-related risk of death, independently from the associated level of alcohol use.

In 2012, a pharmacovigilance report issued by the French Drug Agency pointed out a possible risk of increased suicidal behaviors among AUD subjects (55). This safety signal was never confirmed however. Here again, peer-reviewed studies have highlighted that AUD patients have many psychiatric comorbidities that can trigger suicidal ideations and behaviors, which can thus skew the unadjusted findings that baclofen treatment is associated with an increased risk of suicidal behaviors.

## Discussion

Among the different ADRs induced by baclofen in AUD patients, the most frequent types surely pertain to sedation and the associated symptoms, e.g., drowsiness, dizziness, or confusion. These ADRs are certainly those which raise the most public health concerns in baclofen use among AUD patients, including in the labeling decision. It seems now relatively demonstrated that baclofen immediate dosing is correlated with the risk of occurrence of sedation, as well as with the severity of sedation. However, the main risk factors for severe sedation are clearly the concomitant heavy use of alcohol (21). Similarly, though it has not been studied and thus demonstrated, it is very likely that the concurrent use of other sedative medications, e.g., benzodiazepines, or antipsychotics, or sedative drugs of abuse, e.g., opiates, or cannabis, may also enhance the overall level of sedation among the patients treated with baclofen. The association of baclofen with alcohol use is of course frequent in real-life AUD patients, but other associations with sedative substance or medications are also common, and no longitudinal study has assessed so far the extent to which either baclofen, or other drugs, actually contribute to the overall safety risks of patients.

This specific issue of causality is of particular importance in the case of acute intoxications. Baclofen is rarely the unique drug ingested, and the most frequent situations relate to complex drug mixes, which include potentially lethal drugs such as alcohol, benzodiazepines, or opiates. In practice, no study has ever assessed the respective causality of each molecule in the vital prognosis of the intoxicated patients. It is likely that the ingested dose of baclofen can contribute to the overall severity of acute drug intoxications, but no current evidence suggests that it is a major contributing factor, compared to other sedative drugs or sedative medications. In France, the increasing use of baclofen in AUD patients has led to an increasing number of intoxications involving baclofen (26). AUD patients are particularly exposed to suicide attempts and thus drug intoxications. At this stage, it remains unclear whether baclofen actually enhances the lethal risk related to drug intoxication.

Currently, the efficacy level of baclofen in AUD is still under debate, in particular at high doses (3). This has put the use of baclofen into question by some authors (15, 56). The same conclusion can be drawn of baclofen safety. Consequently, and despite the wide literature published on baclofen efficacy and safety in AUD patients, defining the exact benefit/risk ratio has been considered currently impossible, either by international authors (2), or by the French Alcohol Society (57). In view of these uncertainties, the French Drug Agency had issued a very supervised protocol of use of baclofen for AUD, though this measure was almost not applied on the ground (58).

It has been argued that the clinical trials that have assessed baclofen efficacy were not designed according to the common empirical use of baclofen for AUD, according to which baclofen dosing is a permanent patient-tailored equation which includes the immediate impact of craving and alcohol use on the one hand, and the overall tolerability on the other hand (13). The only study whose protocol followed this dosing scheme was “Bacloville,” a French study that has been announced as positive on efficacy (17), but which is currently still unpublished more than 4 years after the end of the trial. All put together, the scientific evidence on baclofen is confused and unclear. Baclofen efficacy, in particular at high doses, lacks clear evidence. Similarly, baclofen tolerability has been questioned by some pharmacovigilance signals, but no well-designed unbiased study has ever demonstrated that baclofen could be clearly harmful for AUD patients. In this context, a labeling is being examined by the French Drug Agency, and their decision is clearly difficult to guess at this stage.

## Author contributions

BR, NS, and NF conducted the literature search and wrote the manuscript.

### Conflict of interest statement

BR and NS declare having conflicts of interest with Ethypharm.

The remaining author declares that the research was conducted in the absence of any commercial or financial relationships that could be construed as a potential conflict of interest.
